# Development of Maximal Dynamic Strength During Concurrent Resistance and Endurance Training in Untrained, Moderately Trained, and Trained Individuals: A Systematic Review and Meta-analysis

**DOI:** 10.1007/s40279-021-01426-9

**Published:** 2021-03-22

**Authors:** Henrik Petré, Erik Hemmingsson, Hans Rosdahl, Niklas Psilander

**Affiliations:** 1 Department of Physiology, Nutrition and Biomechanics, The Swedish School of Sport and Health Sciences, SE-114 86, PO Box 5626, Stockholm, Sweden; 2 Department of Physical Activity and Health, The Swedish School of Sport and Health Sciences, SE-114 86, PO Box 5626, Stockholm, Sweden

## Abstract

**Background:**

The effect of concurrent training on the development of maximal strength is unclear, especially in individuals with different training statuses.

**Objective:**

The aim of this systematic review and meta-analysis study was to compare the effect of concurrent resistance and endurance training with that of resistance training only on the development of maximal dynamic strength in untrained, moderately trained, and trained individuals.

**Methods:**

On the basis of the predetermined criteria, 27 studies that compared effects between concurrent and resistance training only on lower-body 1-repetition maximum (1RM) strength were included. The effect size (ES), calculated as the standardised difference in mean, was extracted from each study, pooled, and analysed with a random-effects model.

**Results:**

The 1RM for leg press and squat exercises was negatively affected by concurrent training in trained individuals (ES =  – 0.35, *p* < 0.01), but not in moderately trained ( – 0.20, *p* = 0.08) or untrained individuals (ES = 0.03, *p* = 0.87) as compared to resistance training only. A subgroup analysis revealed that the negative effect observed in trained individuals occurred only when resistance and endurance training were conducted within the same training session (ES same session =  – 0.66, *p* < 0.01 vs. ES different sessions =  – 0.10, *p* = 0.55).

**Conclusion:**

This study demonstrated the novel and quantifiable effects of training status on lower-body strength development and shows that the addition of endurance training to a resistance training programme may have a negative impact on lower-body strength development in trained, but not in moderately trained or untrained individuals. This impairment seems to be more pronounced when training is performed within the same session than in different sessions. Trained individuals should therefore consider separating endurance from resistance training during periods where the development of dynamic maximal strength is prioritised.

**Supplementary Information:**

The online version contains supplementary material available at 10.1007/s40279-021-01426-9.

## Key Points

**Table:** 

The main finding of this systematic review and meta-analysis was that concurrent resistance and endurance training had a negative effect on lower-body strength development in trained but not in moderately trained or untrained individuals.	
This impairment seems to be present only when resistance and endurance training are performed within a short interval between each other (< 20 min), that is, within the same training session but not when performed separately (> 2 hours).	

## Introduction

Optimising adaptations from resistance and endurance training are important for general health and elite sports performance. To maintain health in the general population, the current recommendation from the World Health Organisation includes both endurance training for increased activation of the cardiorespiratory system and muscle-strengthening exercise on a weekly basis [1]. Both exercises have the potential to promote adaptation within the skeletal muscle, which preserves functional capacity and metabolic health. From an athletic perspective, few sports are strictly endurance or strength based; instead, a combination of both strength and endurance is required for optimal performance. Including both resistance- and endurance-based exercises in the same or different training sessions within the same periodised training programme is termed *concurrent training* [2]. Because the pioneering study by Hickson, concurrent training has often been associated with attenuated strength development, a phenomenon named “the interference effect” [3]. However, subsequent studies have shown conflicting results; some are in line with those reported by Hickson, showing the negative effect of concurrent training on strength progression [3–9], whereas others are not [10–16].

One explanation for these divergent findings could be that the training status of the participants differed among the studies. In line with this, a recent review proposed that the inhibitory effect of concurrent training on strength development is more prominent in moderately trained and trained individuals [17], possibly because trained individuals have a lower potential for adaptations. Training, therefore, needs to be highly specific to achieve further gains. Even a small interference effect would then be enough to impair the strength development in this population. Furthermore, untrained individuals can obtain adaptations after endurance training that are normally associated with resistance training, such as muscle hypertrophy and increased strength [18–20]. Therefore, it is reasonable to assume that untrained persons might benefit, or at least will not have a disadvantage, from including endurance training in their resistance training programme, whereas this may not be the case for trained individuals.

Further support for this theory becomes evident when categorising studies according to training status. If concurrent training studies with multi-joint outcome measures, such as squat and leg press exercises are selected, most would show no significant negative effect on strength development if the participants are untrained [11–14, 21–23]. In addition, some of these studies even show overlapping adaptations, with the group only performing endurance training having significant gains in strength from pre- to post-test [12, 22]. On the other hand, if the participants are trained, the relationship seems to be the opposite; that is, a clear majority shows an interference effect of endurance training on strength development [4, 6, 8, 9, 24, 25].

Overall, the effect of a concurrent training programme on strength development seems to be influenced by the training status of the participants. However, it is difficult to draw any conclusions from the results of individual studies because most concurrent studies are small and potentially underpowered. The primary aim of the present study was therefore to perform a systematic review and meta-analysis to compile and analyse the results of a large number of concurrent studies to identify whether the earlier reported interference effect is attenuated or augmented by the training status. This was performed by categorising the studies according to training status and comparing the overall effect sizes (ES) of the categories.

The recovery period between the endurance and resistance exercises during concurrent training has been highlighted as a crucial factor for strength development [25, 26]. However, not much research has been conducted on this topic. Robineau et al. found that in trained individuals, the interference effect was stronger when strength and endurance exercises were performed within the same session than when performed with either a 6- or 24-h interval between training sessions [25]. Whether this effect is similar for participants of different training statuses are unclear. The secondary aim of this meta-analysis was; therefore, to perform subgroup analyses to determine whether the recovery period between the endurance and resistance training sessions could influence the potential interference effect. The knowledge derived from this study could be useful for exercise scientists, physiotherapists, coaches, athletes, and other fitness professionals when prescribing concurrent resistance and endurance training programmes.

## Methods

### Search Strategy

A systematic review and meta-analysis was conducted in accordance with the Preferred Reporting Items for Systematic Reviews and Meta-analyses statement guidelines [27]. A search from January 1980 to May 2020 was performed primarily using the PubMed and SPORTDiscus databases. The search strategy used the following combined terms: ‘concurrent strength and endurance training’ OR ‘combined strength and endurance training’ OR ‘simultaneous strength and endurance training’ OR ‘concurrent resistance and endurance training’ OR ‘simultaneous resistance and endurance training’ OR ‘combined resistance and endurance training’ OR ‘cross training strength and endurance’ OR ‘cross training resistance and endurance’. These search terms were used because they were deemed relevant and associated with concurrent training. The title and abstract of the studies identified through the database search were scanned for potential inclusion according to the inclusion criteria. We also manually searched the reference lists of the included papers, whereby eight more studies were included. Studies with insufficient information in the abstract to decide their inclusion or exclusion for final analysis were retrieved for full-text analysis and evaluation. The corresponding authors of the articles were contacted if relevant data were lacking. The search was limited to humans, the English language, and adults.

### Inclusion Criteria

The following inclusion criteria were applied: (1) randomised and nonrandomised original articles, including healthy normal-weight men and women, 18–40 years of age; (2) interventions compared a group performing lower-body resistance and endurance trainings with a group performing identical resistance training only; (3) participants in the intervention and control groups with an equal baseline training status; (4) resistance training programmes performed in at least two sessions per week, including the same exercises as the main outcome measure of the study, with an intensity > 60% of the 1RM or lighter weight to fatigue; (5) endurance training performed as running or cycling at an intensity > 70% of the maximal heart rate in at least 2 sessions per week; and (6) studies reporting changes in maximal strength in leg press or squat exercise. These exercises were chosen because they are valid and reliable 1RM tests for maximal strength [28, 29], are widely used during lower-limb resistance training, and stimulate the major muscle groups in the lower limbs. The rationale for including both randomised and nonrandomised studies was to maximise data for the final analysis. Age and health restrictions were chosen to enable homogeneous groups of subjects and the potential influence of confounding factors. Interventions with equal resistance training programs were specifically chosen to ensure comparable resistance training stimuli for the intervention and control groups. The rationale for only including studies with a training intensity and duration above certain thresholds was to ensure that the training stimulus would be sufficient to evoke adaptations in both maximal strength and cardiorespiratory fitness [30, 31]. We confirmed that studies that used metrics different from the maximal heart rate met the 70% inclusion criteria by scrutinising the training design characteristics of the endurance training and the prescribed intensities. In studies with obvious error or typographical errors in key outcomes, scientific reasoning and comparisons with other equivalent studies were applied as recommended in the Cochrane Handbook for Systematic Reviews of Interventions, specifically to handle situations where authors are not responding to requests to clarify uncertainties [32]. Scientific reasoning enables sound assumptions about what is missing or is a typographical error. In this report scientific reasoning was used to recalculate the standard deviation (SD) to the standard error of the mean (SEM) or vice versa, where it obviously was a typographical error, or to add information missing in the training design.

To classify the training status, we followed recent recommendations suggesting that training status is a consequence of training history/experience (length of time spent regularly performing an activity or exercising) rather than an objective measure such as 1RM or VO_2max_ [33, 34]. Objective measures can be added to make a classification more robust but only if the same tests and standardised procedures have been used. As this requirement differed considerably between the studies included in this analysis, especially for measurements of 1RM, we did not include objective measures in the classification process. To distinguish between physical activity and exercise, we used structure, planning, and repetition (regularity) as recommended by the World Health Organization [1].

The following criteria were used to classify participants as untrained, moderately trained, and trained:

- Untrained: individuals classified as untrained or sedentary by the author or who reported no involvement in regular physical activity for at least 3 months prior to the intervention period.
- Moderately trained: individuals classified as recreationally or physically active but not involved in a regular structured training programme for at least 3 months prior to the intervention period.
- Trained: individuals classified as athletes or individuals who participated in regular structured training programmes for at least 3 months prior to the intervention period.

In two studies, participants classified as recreationally active by the author were classified as trained, as they were involved in systematic resistance and/or endurance training [35, 36]. To enable a subgroup analysis, the studies were further divided into categories based on whether the resistance and endurance trainings were performed during the same session (< 20 min between exercises) or different sessions (> 2 h between sessions). The < 20-min and > 2-h recovery periods were chosen because none of the studies had periods in between these time points, and they well represent what we considered to be training during the same session as compared with training at different sessions. If a study mixed the same session and different sessions, they were placed into a mixed category and were not included in the subgroup analysis. This applied to two studies [8, 22].

### Quality Assessment

The methodological quality of the included studies was assessed with the PEDro Scale [37]. Only moderate- to high-quality studies (PEDro scores 5–10) were included in the meta-analysis. All the studies were rated according to the PEDro scale by two researchers (HP and HR). The result from the quality assessment can be found in the supplementary information (Appendix S1). Fourteen of the studies [3, 4, 10, 11, 14, 15, 21–24, 35, 38–40] provided evidence of moderate quality, and 13 studies [5, 6, 8, 12, 13, 16, 25, 36, 41–45] presented evidence of high quality.

### Statistical Analyses

The mean and SD or confidence interval (CI) was used to present the data. Calculations and analyses were performed with Comprehensive Meta-Analysis (CMA) version 2 (Biostat, Inc., Englewood, NJ, USA). Significance levels were set to p < 0.05. Standard differences in the mean were used to calculate the ES. The threshold for the smallest worthwhile change was set to 0.2. An ES ≥ 0.2 was considered a small effect; ≥ 0.5, a moderate effect; and ≥ 0.8, a large effect [46]. The mean relative change (%) in maximal strength, calculated as the post-training result minus the pre-training result divided by the pre-training value and multiplied by 100, was calculated for both groups (the concurrent resistance and endurance training [CT] group and resistance training only [RT] group). The level of heterogeneity was calculated using the *I*^2^ statistics. The threshold for heterogeneity was set as follows: an *I*^2^ value of 25% was considered low; 50%, moderate; and 75%, high [47]. The data were collected from each study and analysed with a random-effects model, and are presented visually in forest plots. The pooled ES with a 95% CI is presented for each category and was compared between the groups. To calculate the ES in CMA, pre- and post-training data for each group (mean, SD, and N) were used. In five of the included studies, the mean and SD were extracted manually from graphs [3, 4, 6, 14, 38]. Funnel plots stratified by training status were used to quantify potential publication bias. As most studies were small-scale in terms of sample size, we also performed an additional sensitivity analysis using Hedges’ *g* as a complement to standardised difference in means, to evaluate the robustness of our results. A subgroup analysis was performed to examine if the recovery period between the endurance and resistance training sessions could affect the development of maximal strength. To do this, the studies were divided into studies that performed the training in close proximity during the same session (< 20 min apart) and studies that performed the training at different sessions (> 2 h apart).

## Results

### Description of Studies

**Fig.1:**
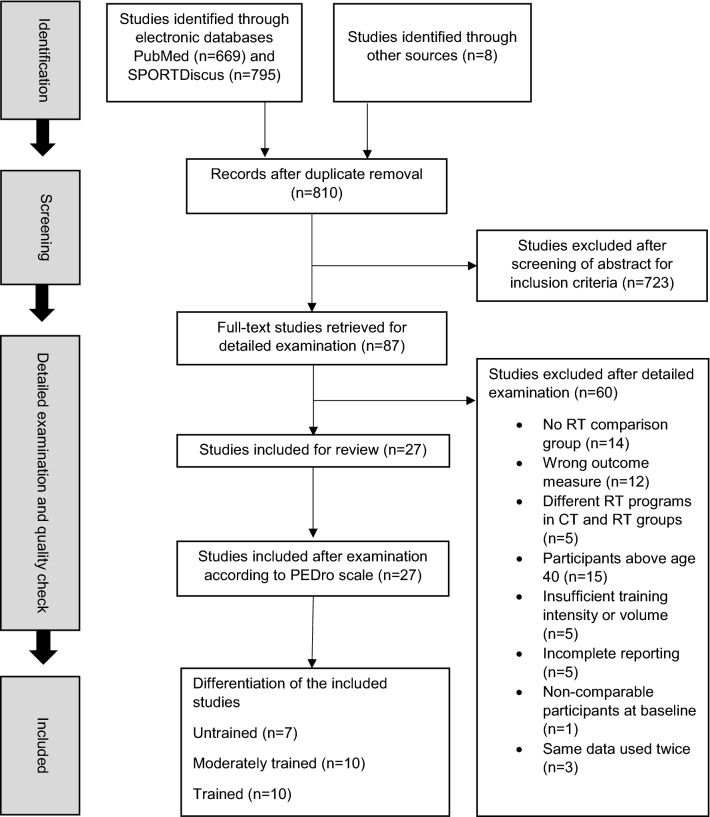
Flowchart diagram of the study screening process. *RT* resistance training, *CT* concurrent training, *n* number of studies

The database search yielded 1464 potential studies for inclusion (Fig. 1). Twenty-seven studies met the inclusion criteria, and were included in the meta-analysis [3–6, 8, 10–16, 21–25, 35, 36, 38–45]. A total of 750 participants were included (523 men and 227 women), aged 20–38 years. Seven studies involved untrained individuals [11–14, 21–23], 10 studies involved moderately trained individuals [3, 5, 10, 15, 16, 38–41, 45], and 10 studies involved trained individuals [4, 6, 8, 24, 25, 35, 36, 42–44]. The corresponding authors of 16 studies were contacted [3–6, 14–16, 21, 22, 25, 36, 38–42] for clarification or missing information via e-mail, of whom five responded with additional information [5, 15, 16, 36, 39]. In two of the included studies, it was not clear in which form the variation (SD and SEM) was reported. Scientific reasoning by comparisons with equivalent studies were then applied to recalculate the variation in the outcomes, as the authors would not respond to requests for clarification [6, 24]. For more detailed information about the participant characteristics, see Table 1.

**Table 1:** Participant characteristics

Study	Group	Participants (*n*)	Age	Body weight (kg)	Sex M (%)	Training status
Craig et al. [21]	CT	12	23.5 ± 1.7ª	75.0 ± 4.6	100	Untrained
	RT	11		74.5 ± 3.0	100	Untrained
Glowacki et al. [12]	CT	16	22.0 ± 2.0	91.6 ± 17.1	100	Untrained
	RT	13	23.0 ± 3.0	72.8 ± 11.9	100	Untrained
Hunter et al. [22]	CT	8	NR	69.4 ± 8.8	62.5	Untrained
	RT	10	NR	64.4 ± 10.1	50	Untrained
Kazior et al. [23]	CT	9	26.0 ± 5.3	77.5 ± 12.1	100	Untrained
	RT	7	28.0 ± 3.7	76.6 ± 6.7	100	Untrained
McCarthy et al. [14]	CT	10	27.3 ± 5.4	82.1 ± 13.6	100	Untrained
	RT	10	27.9 ± 3.8	82.0 ± 13.9	100	Untrained
Mikkola et al. [11]	CT	11	37.0 ± 5.0	88.6 ± 12.9	100	Untrained
	RT	16	38.0 ± 6.0	84.7 ± 15.6	100	Untrained
Volpe et al. [13]	CT	10	20.1 ± 0.9	62.0 ± 9.2	0	Untrained
	RT	8	21.0 ± 1.4	58.7 ± 10.5	0	Untrained
Bell et al. [41]	CT1	8	22.3 ± 3.3ª	73.4 ± 11.6ª	100	Moderately trained
	CT2	5			0	Moderately trained
	RT1	7			100	Moderately trained
	RT2	4			0	Moderately trained
de Souza et al. [16]	CT	11	22.5 ± 3.9	72.9 ± 9.8	100	Moderately trained
	RT	11	25.9 ± 6.4	73.5 ± 16.1	100	Moderately trained
Fyfe et al. [5]	CT1	7	30.8 ± 7.1	85.5 ± 9.8	100	Moderately trained
	CT2	8	29.5 ± 2.1	82.6 ± 10.9	100	Moderately trained
	RT	8	28.6 ± 6.4	86.6 ± 14.0	100	Moderately trained
Hickson [3]	CT	7	26.0	82.2 ± 19.3	71.4	Moderately trained
	RT	8	22.0	75.8 ± 9.6	87.5	Moderately trained
Häkkinen et al. [10]	CT	11	37.0 ± 5.0	88.6 ± 12.9	100	Moderately trained
	RT	16	38.0 ± 5.0	83.9 ± 15.0	100	Moderately trained
Laird et al. [38]	CT	14	20.2 ± 1.5	63.3 ± 9.9	0	Moderately trained
	RT	14	20.4 ± 1.9	62.6 ± 8.2	0	Moderately trained
Lee et al. [15]	CT1	10	24.5 ± 4.7ª	74.9 ± 11.7	100	Moderately trained
	CT2	10		74.3 ± 11.0	100	Moderately trained
	RT	9		75.7 ± 10.7	100	Moderately trained
Shamim et al. [39]	CT	12	26.0 ± 4.0	76.4 ± 10.2	100	Moderately trained
	RT	10	24.0 ± 6.0	75.5 ± 10.3	100	Moderately trained
Silva et al. [45]	CT1	10	22.3 ± 2.1	59.8 ± 6.7	0	Moderately trained
	CT2	11	24.3 ± 5.0	59.0 ± 5.9	0	Moderately trained
	CT3	11	21.6 ± 1.8	60.8 ± 6.5	0	Moderately trained
	RT	12	23.5 ± 2.5	59.2 ± 8.3	0	Moderately trained
Tsitkanou et al. [40]	CT	10	21.8 ± 2.8ª	74.2 ± 9.6ª	100	Moderately trained
	RT	11			100	Moderately trained
Balabinis et al. [24]	CT	7	22.6 ± 2.1	86.1 ± 1.8	100	Trained
	RT	7	22.2 ± 1.0	85.4 ± 1.4	100	Trained
Cantrell et al. [35]	CT	7	26.6 ± 6.6	80.9 ± 11.2	100	Trained
	RT	7	24.7 ± 5.9	78.1 ± 9.7	100	Trained
Chtara et al. [4]	CT1	10	21.4 ± 1.3ª	70.7 ± 6.6	100	Trained
	CT2	10		73.9 ± 6.3	100	Trained
	RT	9		68.9 ± 2.9	100	Trained
Dolezal & Potteiger [42]	CT	10	20.1 ± 1.6ª	72.8 ± 7.6	100	Trained
	RT	10		76.9 ± 7.4	100	Trained
Hennessy & Watson [8]	CT	10	23.4 ± 3.6	80.6 ± 8.6	100	Trained
	RT	9	24.3 ± 3.6	78.9 ± 10.3	100	Trained
Kraemer et al. [6]	CT	9	23.3 ± 3.6	74.2 ± 6.7	100	Trained
	RT	9	24.3 ± 5.1	76.6 ± 14.0	100	Trained
Mirghani et al. [43]	CT	8	21.0 ± 1.1	69.0 ± 6.4	100	Trained
	RT	8	20.5 ± 1.2	67.9 ± 5.0	100	Trained
Panissa et al. [36]	CT	11	24.5 ± 3.7	74.6 ± 6.8	100	Trained
	RT	8	28.7 ± 3.4	77.5 ± 12.9	100	Trained
Robineau et al. [25]	CT1	15	24.3 ± 3.8	85.7 ± 11.5	100	Trained
	CT2	11	28.0 ± 4.5	90.4 ± 9.1	100	Trained
	CT3	12	24.8 ± 3.9	83.5 ± 14.9	100	Trained
	RT	10	25.2 ± 4.4	90.8 ± 14.5	100	Trained
Robineau et al. [44]	CT1	9	25.0 ± 3.7	86.2 ± 10.5	100	Trained
	CT2	10	26.4 ± 3.0	89.3 ± 10.3	100	Trained
	RT	11	27.5 ± 2.5	89.4 ± 14.2	100	Trained

Values are presented as mean ± SD

*RT* resistance training, *RT1* resistance training group 1, *RT2* resistance training group 2, *CT* concurrent training, *CT1* concurrent training group 1, *CT2* concurrent training group 2, *CT3* concurrent training group 3

ªAverage value for all groups, including the control group

### Intervention Characteristics

A summary of training design variables for lower-body strength and endurance training in each study, including recovery between sessions, the sequential order, the frequency and duration of endurance training per session, and the intervention length, is presented in Table 2. The frequency and duration of endurance training per week ranged from 2 to 6 sessions per week and from 12 to 260 min per week. In six of the included studies, information regarding the rest and work durations during intervals was missing. As the authors did not respond with additional information, the endurance duration per session was estimated through scientific reasoning based on the distance that was covered during the endurance training and comparisons with other similar studies [4, 6, 24, 25, 39, 44]. The length of the studies ranged from 6 to 21 weeks. The mean frequency of the endurance training sessions was 2.9 per week for untrained participants, 2.8 for moderately trained participants, and 2.6 for trained participants. The mean duration of the endurance training per session was 37 min for untrained participants, 31 min for moderately trained participants, and 29 min for trained participants. The frequency of strength training ranged from 2 to 5 sessions per week with a mean frequency of 2.9 sessions per week for untrained participants, 2.7 for moderately trained participants, and 2.5 for trained participants. For untrained participants, 4 studies performed endurance training first, and in 3 studies, the intra-session order was not specified. For moderately trained participants, 4 studies performed endurance training first, and 2 performed resistance training first; and in 6 studies, the intra-session order was not specified. For trained participants, 4 studies performed endurance training first, and 5 studies performed resistance training first; and in 3 studies, the intra-session order was not specified.

**Table 2:** Training design characteristics

Study	Group	Exercise prescription	Same or different session (DS, SS or MIX)	Sequential order (E + RT, RT + E or N/A)	Endurance frequency (days/week)	Endurance duration (min session)	Length (weeks)
**Untrained**							
Craig et al. [21]	CT	E: 30–35 min run @ 75% of MHR. RT: Same as below	SS	E + RT	3	33	10
	RT	RT: 3 × 6–8 rep @ 75% of 1RM of LP, LC					10
Glowacki et al. [12]	CT	E: 20–40 min run @ 65–80% of MHR. RT: Same as below	DS	N/A	2–3	25	12
	RT	RT: 3 × 6–10 rep @ 75–85% of 1RM of LP, LC					12
Hunter et al. [22]	CT	E: 20–40 min run @ 75% of MHR. RT: Same as below	MIX	E + RT	4	34	12
	RT	RT: 3 × 7–10 rep @ to failure of BS, LC					12
Kazior et al. [23]	CT	E: 30–60 min bike; 6–8 × 2 min bike @ 60% of VO2max; 95% of VO2max. RT: Same as below	SS	E + RT	3	52	7
	RT	RT: 4–6 × 8–15 rep @ 70% of 1RM or to failure of LP					7
McCarthy et al. [14]	CT	E: 30–50 min bike @ 70% of MHR. RT: Same as below	SS	N/A	3	44	10
	RT	RT: 4 × 5–7 rep @ to failure of BS, KE, LC					10
Mikkola et al. [11]	CT	E: 30–60 min bike; 30–90 min Nordic walking @ below—above AT and above AnT. RT: Same as below	DS	N/A	2	50	21
	RT	RT: 2–4 × 5–15 rep @ 50–80% of 1RM of LP, KE, KF, LAD, LAB					21
Volpe et al. [13]	CT	E: 20–25 min run @ 75% of MHR. RT: Same as below	SS	E + RT	3	23	9
	RT	RT: 2–4 × 4–12 rep @ 60–75% of 1RM of LP, LC, LE					9
		**Mean untrained**			**2.9**	**37**	**12**
**Moderately trained**							
Bell et al. [41]	CT1	E: 30–42 min bike; 4–7 × 3 min on—3 min off bike @ 90% of VO2max. RT: Same as below	DS	N/A	3	41	12
	CT2	E: Same as above. RT: Same as below	DS	N/A	3	41	13
	RT1	RT: 2–6 × 4–12 rep @ 72–84% of 1RM of LP, KF, LE					12
	RT2	RT: Same as above					12
de Souza et al. [16]	CT	E: 15–20 × 60 s on—45 s off run @ 80–100% vVO2max. RT: Same as below	SS	N/A	2	38	8
	RT	RT: 3–5 × 6–12 RM of LP, KE, KF					8
Fyfe et al. [5]	CT1	E: 15–33 min bike @ 80–100% of LT. RT: Same as below	SS	E + RT	3	23	8
	CT2	E: 5–11 × 120 s on—60 s off bike @ 120–150% of LT. RT: Same as below	SS	E + RT	3	22	8
	RT	RT: 3–5 × 4–14 rep @ 65–90% of 1RM of LP, KE, KF, LG					8
Hickson [3]	CT	E: 30–40 min run; 6 × 5 min on—2 min off bike @ 100% of MS; 100% VO2max. RT: Same as below	DS	N/A	6	43	10
	RT	RT: 3–5 × 5 @ 80% of 1RM of BS, KE, KF, LP					10
Häkkinen et al. [10]	CT	E: 30–90 bike or walk @ below-above AT and above AnT. RT: Same as below	DS	N/A	2	50	21
	RT	RT: 3–6 × 3–15 rep @ 50–80% of 1RM of LP, KE					21
Laird et al. [38]	CT	E: 8 × 20 s on—10 s off run @ 110–120% of vVO2max. RT: Same as below	DS	RT + E	3	4	11
	RT	RT: 3–5 × 3–10 rep @ 70–87.5% 1RM of BS, SJ, DL					11
Lee et al. [15]	CT1	E: 8–13 × 120 s on—60 s off bike @ ≈85–97% of Wpeak. RT: Same as below	DS	E + RT	3	30	9
	CT2	E: Same as above. RT: Same as below	DS	RT + E	3	30	9
	RT	RT: 3–4 * 6–12 RM of LP, KE, LG, LC					9
Shamim et al. [39]	CT	E: 4–13 × 10–630 s on and 40–240 s off bike @ 25–110% of MAP. RT: Same as below	DS	N/A	3	42ª	12
	RT	RT: 3–5 × 2–16 rep @ 60–98% of 1RM or to failure of KE, LP					12
Silva et al. [45]	CT1	E: 25–30 min run @ 95% of VT2. RT: Same as below	SS	E + RT	2	26	11
	CT2	E: 20–30 × 60 s on and 60 s off run @ vVO2max. RT: Same as below	SS	E + RT	2	26	11
	CT3	E: 25–30 min bike @ 95% of VT2. RT: Same as below	SS	E + RT	2	26	11
	RT	RT: 2–3 × 8–18 rep @ to failure of LP, KE, KF					11
Tsitkanou et al. [40]	CT	E: 10 × 60 s on and 60 s off bike @ 100% of MAP. RT: Same as below	SS	E + RT	2	20	8
	RT	RT: 4 × 6 rep @ 80–85% of 1RM of LP, HS					8
		**Mean moderately trained**			**2.8**	**31**	**11**
**Trained**							
Balabinis et al. [24]	CT	E: 1–8 rep of 30–90 s run @ 85–90% of MHR. RT: Same as below	DS	E + RT	4	38ª	7
	RT	RT: 1–5 × 3–40 rep @ 40–95% of 1RM of HS, LP					7
Cantrell et al. [35]	CT	E: 4–6 × 20 s bike @ maximal effort. RT: Same as below	DS	N/A	2	16	12
	RT	RT: 3 × 4–6 RM of BS, KE, KF					12
Chtara [4])	CT1	E: 5 × 1/2 of Tmax at MAS run @ 100% of VO2max. RT: Same as below	SS	RT + E	2	25ª	12
	CT2	E: Same as above. RT: Same as below	SS	E + RT	2	25ª	12
	RT	RT: 4–5 × 5–30 rep @ maximum ability of HS, LG, HE					12
Dolezal & Potteiger [42]	CT	E: 25–40 min run @ 65–85% of MHR. RT: Same as below	NR	RT + E	3	35	10
	RT	RT: 3 × 4–15 rep @ to failure BS, KE, LC, LP					10
Hennessy & Watson [8]	CT	E: 20–60 min run; 15–35 min fartlek run @ 70–85% of MHR. RT: Same as below	MIX	N/A	4	35	8
	RT	RT: 2–6 × 6-max rep @ 65–95% of 1RM of BS, HC					8
Kraemer et al. [6]	CT	E: 40 min run; 200–800 m interval run, rest ratio 1:4 to 1:0.5 @ 80–85% of VO2max; 95–100% of VO2max. RT: Same as below	DS	E + RT	4	40ª	12
	RT	RT: 3–5 × 5–10 rep SS, KE, LP, DL					12
Mirghani et al. [43]	CT	E: 16–30 min run @ 65–80% of MHR. RT: Same as below	SS	RT + E	3	23	8
	RT	RT: 2–3 × 6–25 rep @ 55–85% of 1RM of S, LC					8
Panissa et al. [36]	CT	E: 60 on—60 s off intervals to 5 km run @ 100% of MAV. RT: Same as below	SS	E + RT	2	40	12
	RT	RT: 3 × 8–12 RM HS, KE, LC					12
Robineau et al. [25]	CT1	E: 3 × 12 rep of 15 s on—15 s off interval run @ 120% of MAV. RT: Same as below	SS	RT + E	2	30ª	7
	CT2	E: Same as above. RT: Same as below	DS	RT + E	2	30ª	7
	CT3	E: Same as above. RT: Same as below	DS	N/A	2	30ª	7
	RT	RT: 3–4 × 3–10 rep @ 70–90% of 1RM of HS, LP					7
Robineau et al. [44]	CT1	E: 2 × 16–24 rep of 30 s on—30 s off run @ 100% of MAV. RT: Same as below	DS	RT + E	2	21ª	8
	CT2	E: 4–8 × 30 s on and 240 s off run @ All-out. RT: Same as below	DS	RT + E	2	20ª	8
	RT	RT: 3 × 3–10 rep @ 70–90% of 1RM of HS, KE, DL					8
		**Mean trained**			**2.6**	**29**	**9**

*RT* resistance training, *RT1* resistance training group 1, *RT2* resistance training group 2, *DD* different days, *MS* maximal speed, *MHR* maximum heart rate, *MAV* maximal aerobic velocity, *MAP* maximal aerobic power, *Wpeak* peak aerobic power, *VT* ventilatory threshold, *vVO2max* velocity at maximal oxygen uptake, *VO2max* maximal oxygen uptake, *VO2peak* peak oxygen uptake, *Tmax* time to exhaustion, *MAS* maximal aerobic speed, *LT* lactate threshold, *HS* half squat, *PS* parallel squat, *FS* front squat, *LP* leg press, *BS* barbell squat, *KE* knee extension, *KF* knee flexion, *HC* hamstrings curl, *HE* hip extension, *SS* split squat, *LC* leg curl, *LG* lunge, *LAD* leg adduction, *LAB* leg abduction, *SJ* squat jump, *DL* dead lift, *BE* back extension, *LR* lateral raise, *NR* not reported, *DS* different sessions, *SS* same session, *MIX* mix of different session and same session

ªEstimated duration, *N/A* not applicable

The endurance exercise type (interval/continuous/mixed) was 0/6/1 for untrained, 9/6/1 for moderately trained, and 11/2/2 for trained participants. Of the studies, 12 performed concurrent resistance and endurance training within the same session (< 20 min between sessions) [4, 5, 13, 14, 16, 21, 23, 25, 36, 40, 43, 45], 13 performed concurrent resistance and endurance trainings during different sessions (> 2 h between sessions) [3, 6, 10–12, 15, 24, 25, 35, 38, 39, 41, 44], two of the studies mixed performing concurrent resistance and endurance training during the same and different sessions during the training programme [8, 22], and one study did not report whether the trainings were performed in the same or different sessions [42]. With regard to the outcome variables, 15 of the studies measured the maximal dynamic strength with leg press exercise (and two of these also measured squat exercise) [5, 6, 10–13, 15, 16, 21, 23, 24, 39–41, 45], and 14 with squat exercise [3, 4, 8, 14, 22, 24, 25, 35, 36, 38, 40, 42–44].

### Strength Improvement: Concurrent Training Compared with Resistance Training only

The strength improvement for the different interventions included in this meta-analysis is presented in Table 3.

**Table 3:** Effect of concurrent resistance and endurance training compared with resistance training only on maximal strength development

Study	Group	Training status	Exercise	Concurrent training				Resistance training							
				*N*	Pre-training (kg)	Post-training (kg)	Change (%)	*N*	Pre-training (kg)	Post-training (kg)			Change (%)		
Craig et al. [21]	CT	Untrained	Leg press	12	140.9 ± 11.8	147.4 ± 11.3	4.6 ± 2.4	11	136.4 ± 8.7	144.3 ± 9.8			5.8 ± 2.2		
Glowacki et al. [12]	CT	Untrained	Leg press	16	278.0 ± 52.8	387.0 ± 75.3	39.2 ± 2.9	13	221.0 ± 46.1	311.0 ± 62.8			40.7 ± 3.3		
Hunter et al. [22]	CT	Untrained	Squat	8	102.5 ± 38.2	126.9 ± 40.2	23.8 ± 6.1	10	79.5 ± 26.9	110.2 ± 29.4			38.6 ± 6.7		
Kazior et al. [23]	CT	Untrained	Leg press	9	282.0 ± 27.7	367.0 ± 31.2	30.1 ± 1.9	7	292.0 ± 25.7	378.0 ± 37.4			29.5 ± 1.9		
McCarthy et al. [14]	CT	Untrained	Squat	10	102.5ª ± 23.7ª	125.0ª ± 27.7ª	22.0 ± 4.9	10	101.9ª ± 21.7ª	125.0ª ± 23.7ª			23.0 ± 4.7		
Mikkola et al. [11]	CT	Untrained	Leg press	11	171.0 ± 17.0	209.0 ± 24.0	22.2 ± 2.6	16	189.0 ± 27.0	228.0 ± 29.0			20.6 ± 2.8		
Volpe et al. [13]	CT	Untrained	Leg press	10	98.3 ± 13.0	163.2 ± 26.9	66.0 ± 4.5	8	98.4 ± 17.8	155.2 ± 37.3			57.7 ± 5.3		
Bell et al. [41]	CT1	Moderately trained	Leg press	8	276.8 ± 61.7	379.5 ± 45.0	37.1 ± 2.6	7	260.5 ± 78.1	393.6 ± 75.7			51.1 ± 3.4		
	CT2	Moderately trained	Leg press	5	140.0 ± 29.5	257.3 ± 32.4	83.8 ± 4.0	4	151.4 ± 51.8	249.1 ± 151.0			64.5 ± 6.7		
de Souza et al. [16]	CT	Moderately trained	Leg press	11	268.4 ± 47.6	315.7 ± 63.5	17.6 ± 2.8	11	270.3 ± 45.5	320.3 ± 57.0			18.5 ± 2.6		
Fyfe et al. [5]	CT1	Moderately trained	Leg press	7	291.0 ± 68.0	366.0 ± 60.0	25.8 ± 2.7	8	301.0 ± 59.0	412.0 ± 53.0			36.9 ± 2.5		
	CT2	Moderately trained	Leg press	8	299.0 ± 56.0	383.0 ± 60.0	28.1 ± 2.5	8	301.0 ± 59.0	412.0 ± 53.0			36.9 ± 2.5		
Hickson [3]	CT	Moderately trained	Squat	7	85.7ª ± 21.2ª	107.7ª ± 39.3ª	25.0 ± 6.4	8	97.1ª ± 16.2ª	139.1ª ± 25.9ª			44.0 ± 4.7		
Häkkinen et al. [10]	CT	Moderately trained	Leg press	11	171.0 ± 17.0	209.0 ± 24.0	22.2 ± 2.6	16	184.0 ± 29.0	228.0 ± 29.0			23.9 ± 2.9		
Laird et al. [38]	CT	Moderately trained	Squat	14	52.0ª ± 4.0ª	69.3ª ± 4.0ª	33.2 ± 3.8	14	52.0ª ± 9.3ª	70.7ª ± 12.0ª			35.6 ± 6.3		
Lee et al. [15]	CT1	Moderately trained	Leg press	10	328.8 ± 93.5	419.0 ± 106.4	27.4 ± 3.0	9	344.1 ± 100.1	419.4 ± 102.4			21.9 ± 2.9		
	CT2	Moderately trained	Leg press	11	327.3 ± 90.5	412.0 ± 93.7	25.9 ± 2.9	9	344.1 ± 100.1	419.4 ± 102.4			21.9 ± 2.9		
Shamim et al. [39]	CT	Moderately trained	Leg press	12	254.4 ± 64.6	310.8 ± 69.3	24.0 ± 3.2	10	238.3 ± 68.3	309.5 ± 62.4			33.0 ± 3.4		
Silva et al. [45]	CT1	Moderately trained	Leg press	10	100.5 ± 16.3	144.5 ± 23.9	43.8 ± 4.5	12	89.8 ± 16.8	135.3 ± 29.0			50.7 ± 5.3		
	CT2	Moderately trained	Leg press	11	104.2 ± 19.6	152.3 ± 26.3	46.2 ± 4.6	12	89.8 ± 16.8	135.3 ± 29.0			50.7 ± 5.3		
	CT3	Moderately trained	Leg press	11	100.1 ± 21.7	137.3 ± 21.9	37.2 ± 4.7	12	89.8 ± 16.8	135.3 ± 29.0			50.7 ± 5.3		
Tsitkanou et al. [40]	CT	Moderately trained	Leg press	10	259.0 ± 58.5	335.5 ± 44.6	29.5 ± 2.8	11	258.6 ± 53.7	355.9 ± 57.0			37.6 ± 2.9		
	CT	Moderately trained	Squat	10	150.5 ± 20.2	187.0 ± 19.6	24.3 ± 3.0	11	149.5 ± 28.9	191.8 ± 25.5			28.3 ± 3.5		
Balabinis et al. [24]	CT	Trained	Squat	7	102.1 ± 6.2	125.9 ± 13.9	23.3 ± 3.1	7	100.8 ± 6.2	120.2 ± 3.7			19.3 ± 2.2		
	CT	Trained	Leg press	7	220.0 ± 5.3	235.3 ± 10.5	7.0 ± 1.3	7	220.5 ± 6.4	240.7 ± 12.9			8.4 ± 1.4		
Cantrell et al. [35]	CT	Trained	Squat	7	114.4 ± 24.1	147.6 ± 32.7	29.0 ± 4.7	7	115.3 ± 13.9	153.1 ± 19.1			32.8 ± 3.5		
Chtara et al. [4]	CT1	Trained	Squat	10	132.2ª ± 13.0ª	148.4ª ± 6.5ª	12.2 ± 2.4	9	131.6ª ± 6.4ª	155.3ª ± 4.8ª			16.8 ± 1.8		
	CT2	Trained	Squat	10	132.9ª ± 8.4ª	147.7ª ± 8.4ª	11.2 ± 2.2	9	131.6ª ± 6.4ª	155.3ª ± 4.8ª			16.8 ± 1.8		
Dolezal & Potteiger [42]	CT	Trained	Squat	10	100.2 ± 22.8	118.9 ± 21.0	18.7 ± 4.7	10	94.4 ± 22.3	116.1 ± 22.4			23.0 ± 5.0		
Hennessyn & Watson [8]	CT	Trained	Squat	10	112.0 ± 11.1	118.0 ± 9.8	5.4 ± 2.9	9	112.8 ± 16.2	131.7 ± 13.7			16.8 ± 3.4		
Kraemer et al. [6]	CT	Trained	Leg press	9	155.6ª ± 14.8ª	185.9ª ± 24.1ª	19.5 ± 2.8	9	127.8ª ± 25.9ª	168.5ª ± 20.4ª			30.0 ± 3.8		
Mirghani et al. [43]	CT	Trained	Squat	8	97.2 ± 12.7	98.0 ± 15.5	0.8 ± 3.9	8	91.0 ± 15.4	95.8 ± 15.2			5.3 ± 4.3		
Panissa et al. [36]	CT	Trained	Squat	11	113.2 ± 21.8	140.5 ± 25.5	24.1 ± 4.3	8	121.3 ± 23.5	156.5 ± 21.3			29.0 ± 3.9		
Robineau et al. [25]	CT1	Trained	Squat	15	156.0 ± 20.3	182.0 ± 28.5	16.7 ± 3.2	10	152.5 ± 24.6	190.0 ± 38.4			24.6 ± 3.7		
	CT2	Trained	Squat	11	140.0 ± 26.5	184.1 ± 34.0	31.5 ± 3.6	10	152.5 ± 24.6	190.0 ± 38.4			24.6 ± 3.7		
	CT3	Trained	Squat	12	143.3 ± 23.6	180.0 ± 26.4	25.6 ± 3.5	10	152.5 ± 24.6	190.0 ± 38.4			24.6 ± 3.7		
Robineau et al. [44]	CT1	Trained	Squat	9	145.6 ± 17.4	163.3 ± 16.8	12.2 ± 2.8	11	161.4 ± 18.2	187.3 ± 34.1			16.0 ± 3.2		
	CT2	Trained	Squat	10	161.0 ± 18.7	184.0 ± 38.6	14.3 ± 3.3	11	161.4 ± 18.2	187.3 ± 34.1			16.0 ± 3.2		

Pre- and post-training values are presented as mean ± SD

*RT* resistance training, *CT* concurrent training, *CT1* concurrent training group 1, *CT2* concurrent training group 2, *CT3* concurrent training group 3

ªData extracted from graph (mean ± SD)

### Primary Analyses: Training Status

**Fig. 2:**
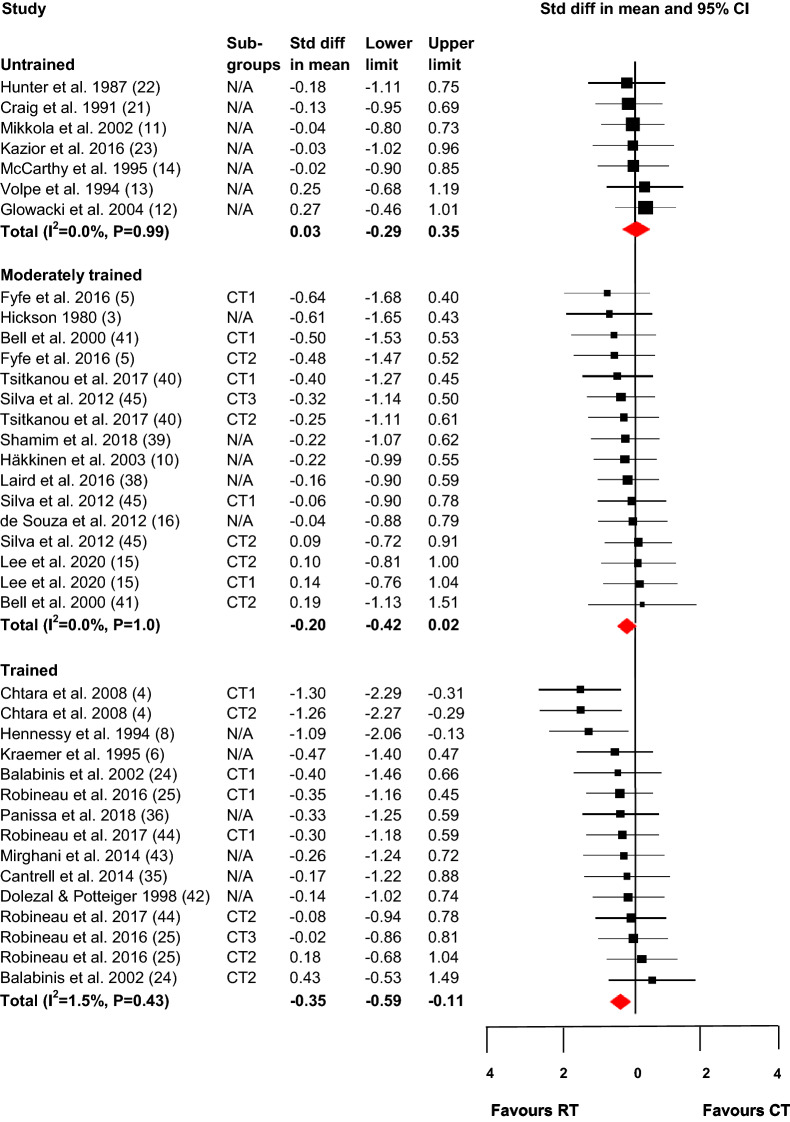
Effect on maximal strength of concurrent resistance and endurance training compared with resistance training only. *CT* concurrent training, *CT1* concurrent training group 1, *CT2* concurrent training group 2, *CT3* concurrent training group 3, *RT* resistance training, *N/A* not applicable (only one concurrent training group). The shaded square represents the estimated intervention effect for each study, and the horizontal line represents the 95% CI. The size of the shaded square represents the relative weight of the study in the meta-analysis. The shaded diamond represents the pooled standard difference in mean. *P* values for the effect difference in each category: untrained, *P* = 0.87; moderately trained, *P* = 0.08; trained, *P* < 0.01

The effect of concurrent resistance and endurance training compared with that of resistance training only on the maximal strength for the three categories, untrained, moderately trained, and trained, is shown in Fig. 2. For untrained and moderately trained participants, there were no significant negative effects of concurrent resistance and endurance training as compared with resistance training alone (ES = 0.03, 95% CI  – 0.29 to 0.35; *p* = 0.87 and ES =  – 0.20, 95% CI  – 0.42 to 0.02; *p* = 0.08). For trained individuals, there was a small significant negative effect favouring resistance training alone compared with concurrent resistance and endurance training (ES =  – 0.35, 95% CI  – 0.59 to  – 0.11; *p* < 0.01). There were no indications of heterogeneity in terms of ES as follows: untrained, *I*^2^ = 0, *p* = 0.99; moderately trained, *I*^2^ = 0, *p* = 1.0; and trained, *I*^2^ = 1.5, *p* = 0.43. The relative weight contributions of the included studies were evenly distributed.

### Subgroup Analyses: Same Session Compared with Different Sessions

**Fig. 3:**
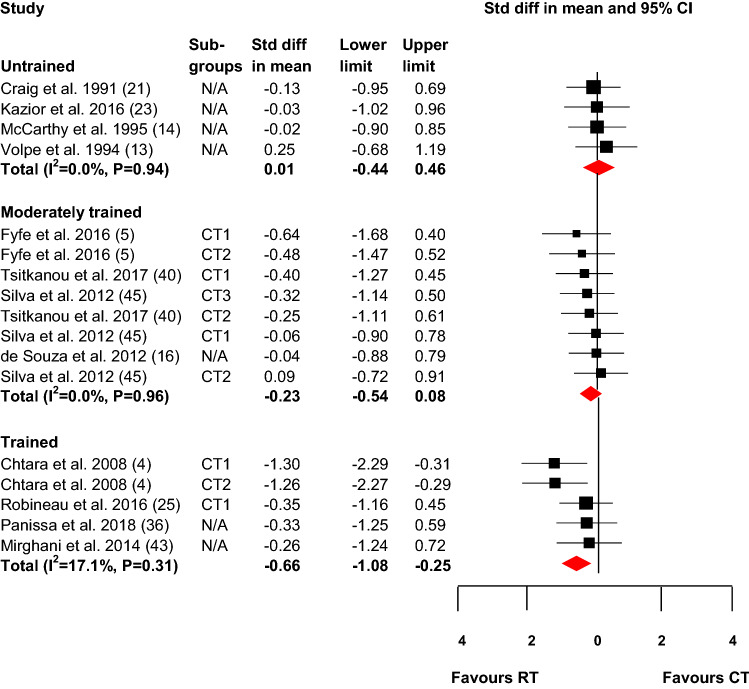
Effect on maximal strength of same session concurrent resistance and endurance training compared with resistance training only. *CT* concurrent training, *CT1* concurrent training group 1, *CT2* concurrent training group 2, *CT3* concurrent training group 3, *RT* resistance training, *N/A* not applicable (only one concurrent training group). The shaded square represents the estimated intervention effect for each study, and the horizontal line represents the 95% CI. The size of the shaded square represents the relative weight of the study in the meta-analysis. The shaded diamond represents the pooled standard difference in mean. *P *values for the effect difference in each category: untrained, *P* = 0.98; moderately trained, *P* = 0.14; trained, *P* < 0.01

**Fig. 4:**
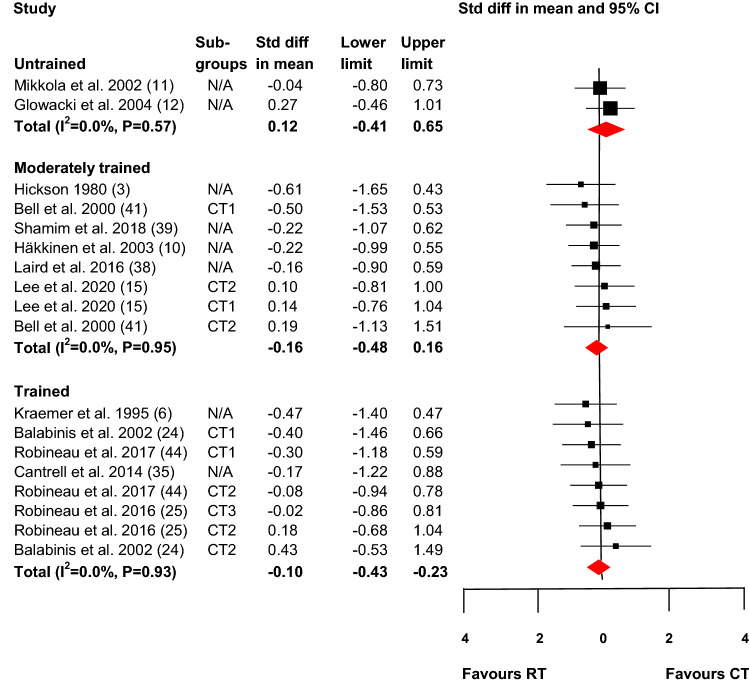
Effect on maximal strength of different session concurrent resistance and endurance training compared with resistance training only. *CT* concurrent training, *CT1* concurrent training group 1, *CT2* concurrent training group 2, *CT3* concurrent training group 3, *RT* resistance training, *N/A* not applicable (only one concurrent training group). The shaded square represents the estimated intervention effect for each study, and the horizontal line represents the 95% CI. The size of the shaded square represents the relative weight of the study in the meta-analysis. The shaded diamond represents the pooled standard difference in mean. *P* values for the effect difference in each category: untrained, *P* = 0.65; moderately trained, *P* = 0.32; trained, *P* = 0.55

A subgroup analysis was performed to compare the ES between studies that performed resistance and endurance trainings within the same session (< 20 min between sessions; Fig. 3) or during different sessions (> 2 h between sessions; Fig. 4). For untrained and moderately trained individuals, there was no significant difference in effect between conducting same session concurrent resistance and endurance training compared with conducting resistance training alone (ES = 0.01; 95% CI  – 0.44 to 0.46; *p* = 0.98 and ES =  – 0.23, 95% CI  – 0.54 to 0.08, *p* = 0.14). However, for trained individuals, the results showed a moderately negative effect favouring resistance training alone compared with conducting resistance and endurance training within the same training session (ES =  – 0.66, 95% CI  – 1.08 to  – 0.25, *p* < 0.01). Low and nonsignificant heterogeneities among the studies were observed for the untrained, moderately trained, and trained individuals (*I*^2^ = 0, *p* = 0.94; *I*^2^ = 0, *p* = 0.96; and *I*^2^ = 17.1, *p* = 0.31, respectively).

Training during different sessions (> 2 h between sessions; Fig. 4) showed no significant difference in effect between concurrent resistance and endurance training and resistance training alone for any of the training status categories (untrained, ES = 0.12, 95% CI  – 0.41 to 0.65, *p* = 0.65; moderately trained, ES =  – 0.16, 95% CI  – 0.48 to 0.16, *p* = 0.32; and trained, ES =  – 0.10, 95% CI  – 0.43 to 0.23, *p* = 0.55). There were no indications of heterogeneity in terms of ES as follows: untrained, *I*^2^ = 0, *p* = 0.57; moderately trained, *I*^2^ = 0, *p* = 0.95; and trained, *I*^2^ = 0, *p* = 0.93.

The stratified funnel plots for training status showed no detectable differences among the groups (data not shown). We also performed a sensitivity analysis using Hedges’ *g* in our main analysis of training category (which may be less sensitive to small sample sizes than the standardised difference in means), but these findings did not materially differ from our main results (data not shown).

## Discussion

### Summary

This is the first systematic review and meta-analysis to investigate whether training status can influence the development of the maximal dynamic strength during concurrent training. The results show that adding endurance training to a resistance training programme impairs the development of the lower-body maximal strength in trained individuals, but not in moderately trained or untrained individuals. However, it is likely that some moderately trained individuals also experience a negative effect of concurrent training, as the results showed a trend for impaired strength development for this category (ES =  – 0.2 and *p* = 0.08). Furthermore, the strength impairment observed for trained individuals seems to be more pronounced when training was performed within the same session than when performed in different sessions.

### Potential Mechanisms

The present results are in line with those of two recent reviews that proposed that the negative effect of adding endurance to resistance training primarily manifests in trained individuals [17, 33]. A possible explanation for this difference between untrained and trained individuals might be that trained individuals have less potential for adaptations and need more specific training to obtain further performance improvements [48, 49]. In line with this are recent findings showing that block periodisation is superior to a mixed training approach for strength development in athletes [50, 51]. The molecular mechanisms behind this are not well understood, but studies that examined the acute response after resistance exercise showed that trained muscles have a blunted expression of several genes and proteins involved in the anabolic adaptation process as compared with untrained muscles [52–54]. Moreover, it was recently shown that mTORC1, a major regulator of muscle hypertrophy, is negatively affected by concurrent training in trained but not in untrained individuals [53], and some studies even showed an enhanced molecular response and hypertrophy in untrained and moderately trained participants after concurrent training [23, 52, 55]. Adding endurance training to a resistance training protocol may therefore, under some circumstances, be beneficial for a less trained population.

Another potential explanation for the impaired response observed in the trained category could be a reduced quality of the performed resistance training because of fatigue. Endurance training can lead to acute fatigue and accumulated fatigue over time (over-reaching), leading to reduced intensity or volume of the resistance training performed [56, 57]. Trained individuals have higher aerobic work capacity than untrained individuals and might therefore exhaust themselves more during endurance training, especially during self-regulated high-intensity endurance training. This could potentially lead to greater fatigue and reduced performance during subsequent resistance training sessions. A higher overall workload might also increase the risk of spending more time in a catabolic state, which would be negative for muscle adaptations, especially if endurance and resistance training are performed in close proximity [33]. In addition, studies have shown that trained individuals have a higher potential for voluntary activation of their muscles [58, 59]. This will result in recruitment of a larger proportion of the muscles during a 1RM test, more weight lifted, and a higher relative mechanical tension when training with a load related to this test (for example 80% of 1RM). They might therefore, be able to stress their muscles more during resistance training and consequently need more recovery between the training sessions than less trained individuals [60]. To summarise, the higher overall load/stress from the resistance and endurance training for trained individuals could potentially enhance the interference effect by reducing the quality of the resistance training and blunting the anabolic response.

### Conflicting Variables

There are several variables other than the training status that might affect the outcomes of a concurrent training programme. Previous reviews on the topic have identified the following as important: the recovery period between the resistance and endurance training sessions, the sequential order of the endurance and resistance trainings (i.e. endurance before or after resistance), and the frequency, duration, intensity, and modality (i.e. cycling, running, etc.) of the endurance training [61–65].

#### Recovery Period (Same Compared with Different Sessions of Concurrent Training)

To date, not much research has been conducted on how different recovery periods between resistance and endurance sessions affect strength adaptations during concurrent training. Robineau and colleagues showed that concurrent training impaired strength development in trained individuals when resistance and endurance training were performed within the same session but not when performed during different sessions [25]. It is not known if this also holds true for moderately trained and untrained individuals. Furthermore, this question has not been studied to any significant extent. Therefore, we performed a sub-analysis to assess the effect of different recovery periods (same vs. different sessions) on lower-body strength for all three categories. The result for the trained category was in line with the previous finding reported by Robineau et al. that showed negative effect when resistance and endurance training were performed within the same training session (< 20 min apart) but not when performed during different sessions (> 2 h apart) [25]. Interestingly, this was not the case for moderately trained and untrained individuals. The results from these two categories showed similar adaptations after concurrent training in the same session compared with different sessions. Even though there was a clear interference effect on strength development in the trained group when endurance and resistance training were performed in close proximity, it is important to point out that this was largely driven by two interventions from the same study [4] and more work is needed to confirm our findings.

#### Sequential Order (i.e. Endurance Before or After Resistance Training)

Two previous reviews have suggested that it may be more beneficial to perform resistance training before endurance training than vice versa for lower-body strength adaptations [61, 62]. This could therefore be a confounding factor in the present analysis if the sequential order was considerably different among our categories. However, this was not the case, as the sequential order of endurance and resistance training was relatively similarly distributed in the three categories. Interestingly, a recent study did not detect any difference in maximal strength development, although some negative effects were noted for power, between concurrent training modalities performed in different orders when the two training sessions were separated by 3 h of rest [15]. Thus, it could be that the sequential order is only important if insufficient recovery is presented between the resistance and endurance training sessions. However, more research is needed to confirm this.

#### Frequency and Duration

In an earlier meta-analysis, Wilson et al. proposed that impaired strength development during concurrent resistance and endurance training might be linked to the frequency and duration of the endurance training performed [64]. They found that strength improvements negatively correlated with increased endurance exercise duration (when increased from 20–30 to 50–60 min/day) and frequency (when increased from 1 to 5 sessions/wk). In addition, Jones et al. investigated different endurance training frequencies with concurrent resistance training for resistance-trained individuals and found that endurance training three times per week was more negative for strength development than training endurance one time per week [66]. However, in the present study, the average frequency and duration of the endurance training were similar among the categories (untrained, 2.9 sessions/wk and 37 min/sessions; moderately trained, 2.8 sessions/wk and 31 min/session; and trained, 2.6 sessions/wk and 29 min/session). Therefore, the frequency and duration, that is, the volume of the endurance training, could not explain the difference between the three categories observed here.

#### Intensity

As mentioned earlier, interval training (i.e. high-intensity training) was more frequently used in the training programmes of those in the trained and moderately trained categories than of those in the untrained category (trained, 11/15; moderately trained, 9/16; and untrained, 0/7). Difference in intensity could potentially explain the larger negative effect of endurance training observed in these categories because high-intensity interval training (HIIT) will recruit a larger proportion of the high-threshold motor units [67] that are also recruited during resistance training [68]. This could potentially enhance the interference effect within this fibre pool, particularly if the endurance and resistance training are executed in close proximity [69]. In the present study, only the moderately trained category included enough studies that performed HIIT and continuous training for an additional subanalysis to study the influence of intensity. This analysis did not show any difference in effect between HIIT and continuous training on the development of maximal strength (data not shown). Therefore, it does not support the theory that the interference effect is enhanced by high-intensity endurance training. In addition, a recent study by Fyfe et al. has shown that HIIT does not affect strength development differently than moderate-intensity continuous training [5] and a review by Sabag et al. showed that resistance training combined with HIIT is an efficient training method for developing maximal strength [63]. However, it is important to highlight that both intervention groups in the study by Fyfe et al. had compromised strength development as compared with the strength training group [5] and in the review by Sabag et al. the authors combined participants across a diverse range of training backgrounds, ranging from untrained to trained individuals [63]. More research is required to determine how endurance exercise intensity affects strength adaptations in individuals with different training statuses. To summarise, although the present subgroup analysis on moderately trained individuals did not show any difference between HIIT and continuous training on the development of strength, it cannot be ruled out that endurance training intensity could have affected our findings.

#### Modality

Most interventions in the trained category used running as the endurance exercise modality (14/15), whereas in the untrained and moderately trained categories, the modality was more evenly distributed between running/cycling (4 running and 3 cycling in the untrained category and 5 running and 9 cycling in the moderately trained category). Wilson et al. suggested that running might have more of a negative effect on strength improvements than cycling, and the large numbers of running interventions in our trained category may therefore be a confounding factor. However, when comparing the cycling study in this category with the running studies, no differences in strength development were apparent. In the review by Sabag et al. it was suggested that cycling might even have a more negative effect on strength development than running [63]. These divergent findings are probably explained by the differences in inclusion criteria and studies included in the analysis. In addition, they were performed approximately 6 years apart. In fact, the meta-analysis by Wilson et al. and Sabag et al. only had two studies in common. Therefore, it cannot be excluded that the endurance exercise modality has influenced our findings. More studies examining this issue are warranted, especially in trained and highly trained individuals.

### Strengths, Limitations and Future Research

The main strength of the present meta-analysis was that we conducted a comprehensive and well-powered analysis that included a large number of studies, for which we also performed sensitivity and subgroup analyses to clarify the robustness of our findings. However, we identified the following challenges: most studies were short in length (< 12 weeks) and/or had a relatively low number of participants (*n* < 10). In addition, there were large variations in how the concurrent training programmes were constructed, and the training status of the participants was not always clearly explained. Despite this, the risk that the studies were misclassified is low because most uncertainties were sorted out by correspondence with the authors. It is important to point out that this study was limited to the categorisation of untrained, moderately trained, and trained individuals and did not include a highly trained category due to the limited number of studies. Hence, we suggest that future studies should include highly trained individuals with a long history of structured training (> 5 years) who are skilled in performing resistance training with complex, multi-joint resistance exercises. In addition, if the above-mentioned criteria are fulfilled, it would be valuable to add quantifiable measures of physical capacity to assure certain strength and endurance levels. Furthermore, the findings of this study are limited to dynamic, multi-joint strength measures, and cannot be generalised to other strength outcomes, such as maximal voluntary contractions in isometric or isokinetic exercises. Future studies should, therefore, focus on examining if these results are transferrable to a more trained population and valid for strength outcomes that are less dependent on technique and inter-muscular coordination. There is also a lack of well-powered studies and studies that examined the long-term effects of concurrent training (> 12 weeks), as well as studies comparing the effects of different endurance training modalities, that is, cycling and running, on strength development. Finally, the lack of research on women, especially trained women, is a clear limitation in the literature which certainly is an area that should be further explored in future studies.

## Conclusion

This meta-analysis shows that concurrent resistance and endurance training has a negative effect on lower-body strength development in trained but not in moderately trained or untrained individuals. The impairment observed in the trained category seems to be present only when resistance and endurance exercises were performed within a short time of each other (< 20 min), that is, within the same training session, but not when performed separately (> 2 h). Trained individuals should therefore consider separating endurance from resistance training, with > 2 h, during periods when strength development should be maximised. A concurrent training programme for untrained or moderately trained individuals can be based on more practical considerations or personal preferences rather than trying to keep resistance separated from endurance training. It is important to acknowledge that even though the strength development was impaired in the trained category, the impairment was moderate. Therefore, athletes with limited time may train for resistance and endurance within the same training sessions and still obtain appropriate increases in lower-body maximal dynamic strength.

## Supplementary Information

Below is the link to the electronic supplementary material.

Supplementary file1 (DOCX 32 KB)
