# Supplementary material for: Development of Maximal Dynamic Strength During Concurrent Resistance and Endurance Training in Untrained, Moderately Trained, and Trained Individuals: A Systematic Review and Meta-analysis
Source: Sports Med. 2021 Mar 22;51(5):991–1010. doi: 10.1007/s40279-021-01426-9 (PMC8053170; doi:10.1007/s40279-021-01426-9)
Supplement: Supplementary file 1 [file 40279_2021_1426_MOESM1_ESM.docx]

Appendix S1. PEDro quality assessment of the included studies.

| **Study** | **1** | **2** | **3** | **4** | **5** | **6** | **7** | **8** | **9** | **10** | **11** | |
|---|---|---|---|---|---|---|---|---|---|---|---|---|
| **Balabinis et al. 2003 (24)** | 1 | 0 | 0 | 1 | 0 | 0 | 0 | 1 | 1 | 1 | 1 | |
| **Bell et al. 2000 (41)** | 1 | 1 | 0 | 1 | 0 | 0 | 0 | 1 | 1 | 1 | 1 | |
| **Cantrell et al. 2014 (35)** | 1 | 0 | 0 | 1 | 0 | 0 | 0 | 1 | 1 | 1 | 1 | |
| **Chtara et al. 2008 (4)** | 1 | 0 | 0 | 1 | 0 | 0 | 0 | 1 | 1 | 1 | 1 | |
| **Craig et al. 1991 (21)** | 1 | 0 | 0 | 1 | 0 | 0 | 0 | 1 | 1 | 1 | 1 | |
| **de Souza et al. 2013 (16)** | 1 | 1 | 0 | 1 | 0 | 0 | 0 | 1 | 1 | 1 | 1 | |
| **Dolezal & Potteiger 1998 (42)** | 1 | 1 | 0 | 1 | 0 | 0 | 0 | 1 | 1 | 1 | 1 | |
| **Fyfe et al. 2016 (5)** | 1 | 1 | 0 | 1 | 0 | 0 | 0 | 1 | 1 | 1 | 1 | |
| **Glowacki et al. 2004 (12)** | 1 | 1 | 0 | 1 | 0 | 0 | 0 | 1 | 1 | 1 | 1 | |
| **Hennessy & Watson 1994 (8)** | 1 | 1 | 0 | 1 | 0 | 0 | 0 | 1 | 1 | 1 | 1 | |
| **Hickson 1980 (3)** | 1 | 0 | 0 | 1 | 0 | 0 | 0 | 1 | 1 | 1 | 1 | |
| **Hunter et al. 1987 (22)** | 1 | 0 | 0 | 1 | 0 | 0 | 0 | 1 | 1 | 1 | 1 | |
| **Häkkinen et al. 2003 (10)** | 1 | 0 | 0 | 1 | 0 | 0 | 0 | 1 | 1 | 1 | 1 | |
| **Lee et al. 2020 (15)** | 1 | 0 | 0 | 1 | 0 | 0 | 0 | 1 | 1 | 1 | 1 | |
| **Kazior et al. 2016 (23)** | 1 | 0 | 0 | 1 | 0 | 0 | 0 | 1 | 1 | 1 | 1 | |
| **Kraemer et al. 1995 (6)** | 1 | 1 | 0 | 1 | 0 | 0 | 0 | 1 | 1 | 1 | 1 | |
| **Laird et al. 2016 (38)** | 1 | 0 | 0 | 1 | 0 | 0 | 0 | 1 | 1 | 1 | 1 | |
| **McCarthy et al. 1995 (14)** | 1 | 1 | 0 | 1 | 0 | 0 | 0 | 0 | 1 | 1 | 1 | |
| **Mikkola et al. 2012 (11)** | 1 | 0 | 0 | 1 | 0 | 0 | 0 | 1 | 1 | 1 | 1 | |
| **Mirghani et al. 2014 (43)** | 1 | 1 | 0 | 1 | 0 | 0 | 0 | 1 | 1 | 1 | 1 | |
| **Panissa et al. 2018 (36)** | 1 | 1 | 0 | 1 | 0 | 0 | 0 | 1 | 1 | 1 | 1 | |
| **Robineau et al. 2016 (25)** | 1 | 1 | 0 | 1 | 0 | 0 | 0 | 1 | 1 | 1 | 1 | |
| **Robineau et al. 2017 (44)** | 1 | 1 | 0 | 1 | 0 | 0 | 0 | 1 | 1 | 1 | 1 | |
| **Shamim et al. 2018 (39)** | 1 | 0 | 0 | 1 | 0 | 0 | 0 | 1 | 1 | 1 | 1 | |
| **Silva et al. 2012 (45)** | 1 | 1 | 0 | 1 | 0 | 0 | 0 | 1 | 1 | 1 | 1 | |
| **Tsitkanou et al. 2017 (40)** | 1 | 0 | 0 | 1 | 0 | 0 | 0 | 1 | 1 | 1 | 1 | |
| **Volpe et al. 1993 (13)** | 1 | 1 | 0 | 1 | 0 | 0 | 0 | 1 | 1 | 1 | 1 | |

1, criterion achieved; 0, criterion not achieved.

**Key: The numbers refer to the questions below**

1. Eligibility critera were specified (does not count toward score)
2. Subjects were randomly allocated to groups
3. Allocation was concealed
4. The groups were similar at baseline
5. There was blinding of all subjects
6. There was blinding of all therapists
7. There was blinding of all assessors
8. Adequate follow-up of at least 85%
9. Intention to treat analysis
10. Between group comparisons reported
11. Point estimates and variability of outcomes provided
